# Comparison of analgesic efficacy of different local anesthetic volumes for erector spinae plane block in thoracotomy patients; a prospective randomized trial

**DOI:** 10.1186/s12871-023-02004-4

**Published:** 2023-02-06

**Authors:** Musa Zengin, Hilal Sazak, Ramazan Baldemir, Gulay Ulger, Dilara Arican, Oya Kaybal, Ali Alagoz

**Affiliations:** University of Health Sciences, Ankara Atatürk Sanatorium Training and Research Hospital, Anesthesiology and Reanimation Clinic, Kuşcağız Mah. Sanatoryum Cad. No: 271, Ankara, Turkey

**Keywords:** Acute pain, Erector spinae plane block, Local anesthetic volume, Postoperative pain, Thoracotomy

## Abstract

**Background:**

Erector spinae plane block (ESPB) is a thoracic wall block that has been used frequently in recent years. It was aimed to compare the analgesic efficacy of bupivacaine in different volumes for ESPB in patients undergoing thoracotomy.

**Methods:**

Patients who were in the age range of 18 to 65 years, ASA I–III, had a body mass index (BMI) of 18–30 kg/m^2^ and were undergoing thoracotomy were included in the study. Patients were assigned to ESPB with 30 ml 0.25% bupivacaine (Group-1) or ESPB with 20 ml 0.25% bupivacaine (Group-2) groups according to the analgesia protocol. In the postoperative care unit, intravenous morphine was administered via a patient-controlled analgesia pump for 24 h. A paracetamol dose of 1 g every 8 h and a dexketoprofen dose of 50 mg twice daily were administered iv for multimodal analgesia.

**Results:**

Visual analog scale (VAS) resting scores, the 1^st^ (*p* = 0.001), 2^nd^ (< 0.001), 4^th^ (< 0.001), 8^th^ (< 0.001), 16^th^ (< 0.010), 24^th^ (< 0.044), and 48^th^ (< 0.005)-hour VAS resting results were found to be statistically significantly higher in the 20 ml group than the 30 ml group. VAS cough scores were statistically significantly higher in the 20 ml group at the 1^st^ (< 0.003), 2^nd^ (< 0.001), 4^th^ (< 0.001), 8^th^ (< 0.001), 16^th^ (< 0.004), 24^th^ (< 0.031), and 48^th^ (< 0.009)-hour. Morphine consumption, and additional analgesic use were found to be statistically significantly higher in the 20 ml group than in the 30 ml group (*p* < 0.001, *p* = 0.001, respectively). There was no statistically significant difference between the groups in terms of side effects (*p* > 0.05).

**Conclusions:**

The results of ESPB applied with 20 ml and 30 ml of local anesthetic before the surgical incision in thoracotomy patients showed that the use of 30 ml of local anesthetic provided more effective analgesia. In addition, similar side-effect rates show that 30 ml of local anesthetic can be used safely.

## Background

Thoracotomy is one of the most painful surgical procedures [1]. An effective analgesic administration accelerates recovery, contributes to the early mobilization of patients, and reduces hospital stay [2, 3]. Thoracic epidural analgesia (TEA) is the most commonly used and still accepted gold standard method in the treatment of post-thoracotomy pain [4]. However, serious complications such as dural puncture, sympathetic block, spinal hematoma, and epidural abscess can also be seen due to epidural application [5]. Thoracic paravertebral block (TPVB) application has also been used in recent years due to the lower incidence of side effects such as hypotension, urinary retention, and nausea and vomiting compared to TEA [4, 6]. With the widespread use of ultrasound (US) in recent years, different thoracic wall block techniques, which are claimed to cause fewer complications, have begun to be applied [7]. The erector spinae plane block (ESPB), one of these blocks, has been used as a part of multimodal analgesia in recent years [8]. In ESPB, first described by Forrero et al. in 2016, it is aimed to treat the postoperative pain of the thoracoabdominal region by injecting a local anesthetic into the interfacial area under the erector spinae muscle [9, 10]. ESPB creates an effect that covers the posterior and lateral thorax by affecting the dorsal rami and branches of the spinal nerves [11, 12]. The risk of complications is less compared to TEA and paravertebral block, as the targeted points are far from the pleura and neuraxial plane while blocking [13].

The hypothesis of this study is that the application of ESPB with 30 ml of local anesthetic after thoracotomy/lobectomy would provide more effective analgesia. The aim of this study is to compare the analgesic efficacy of 0.25% bupivacaine in different volumes for ESPB in patients undergoing thoracotomy.

## Materials and methods

### Study design and patients

The study was conducted with a randomized, prospective, single-blind design after obtaining approval from the Ankara City Hospital Ethical Committee (E.Kurul-E1-21–1964, 25/08/2021). The trial was registered on www.clinicaltrials.gov (https://clinicaltrials.gov/) under the identifier NCT05083845 on 19/10/2021.

After obtaining written informed consent, patients who were in the age range of 18 to 65 years, were assigned American Society of Anesthesiologists (ASA) physical status classifications of I–III, had a body mass index (BMI) of 18–30 kg/m^2^ and were undergoing thoracotomy/lobectomy in the period between August 2021 and June 2022 at a high-volume tertiary thoracic surgery center were included in the study. During the preoperative evaluation, the patients were informed about pain assessment and patient-controlled analgesia (PCA). Patients with preoperative acute or chronic pain and a history of opioid therapy were excluded. Moreover, patients with bleeding disorders, infection at the injection site, or allergy to local anesthetics and patients who underwent emergency surgery, and pleurodesis were excluded from the study.

### Randomization and grouping

Patients were randomly assigned to two groups, 30 each, with 1:1 allocation ratio using computer-generated random numbers. The numbers were concealed in sealed 60 opaque envelopes. Either 30 ml of local anesthetic or 20 ml of local anesthetic was written inside each envelope as the volume for each participant. A blinded anesthetist (an anesthetist who did not participate in the study or data collection) used random numbers to assign patients to their groups. Then the procedure was explained to the patient whether 30 ml of local anesthetic or 20 ml of local anesthetic was performed to him/her. Group I included those to receive 30 ml of local anesthetic and group II was to receive 20 ml of local anesthetic.

### Outcomes

In this study, the primary outcome was determined as visual analog scale (VAS) scores at rest and coughing. The secondary outcomes of this study were postoperative morphine consumptions, the requirement of rescue analgesics, and side effects.

### General anesthesia

Patients were monitored in the operating room by ASA standards. Patients were administered 0.03 mg/kg midazolam for premedication intravenously (iv). Following preoxygenation, anesthesia was induced with 2 mg/kg propofol, 1.5 mcg/kg fentanyl, and 0.1 mg/kg vecuronium. Patients were intubated with a left-sided double-lumen endobronchial tube and the position of the double-lumen endobronchial tube was checked by fiberoptic bronchoscopy. Arterial cannulation was applied to all patients after general anesthesia to perform arterial pressure monitorization and arterial blood gas analyses. Anesthesia was maintained by administering sevoflurane in oxygen and air mixture and by administering remifentanil infusion at a dose of 0.01–0.20 mcg/kg/min. An increase or decrease in the rate of remifentanil infusion was adjusted to hemodynamic parameters. Before the commencement of the surgical procedure, blocks were performed under US guidance. Thoracotomy was performed with the posterolateral technique in a clinic where frequent thoracic surgery and thoracotomy were performed. After thoracotomy, 2 chest tubes were placed in the patients. The first of the chest tubes is placed in the posterior axillary line and the seventh/eighth intercostal space, while the second is placed in the anterior axillary line and the sixth/seventh intercostal space. At the end of the operation, all patients were extubated and transferred to the surgical intensive care unit in spontaneous breathing.

### Block procedures

**Fig. 1:**
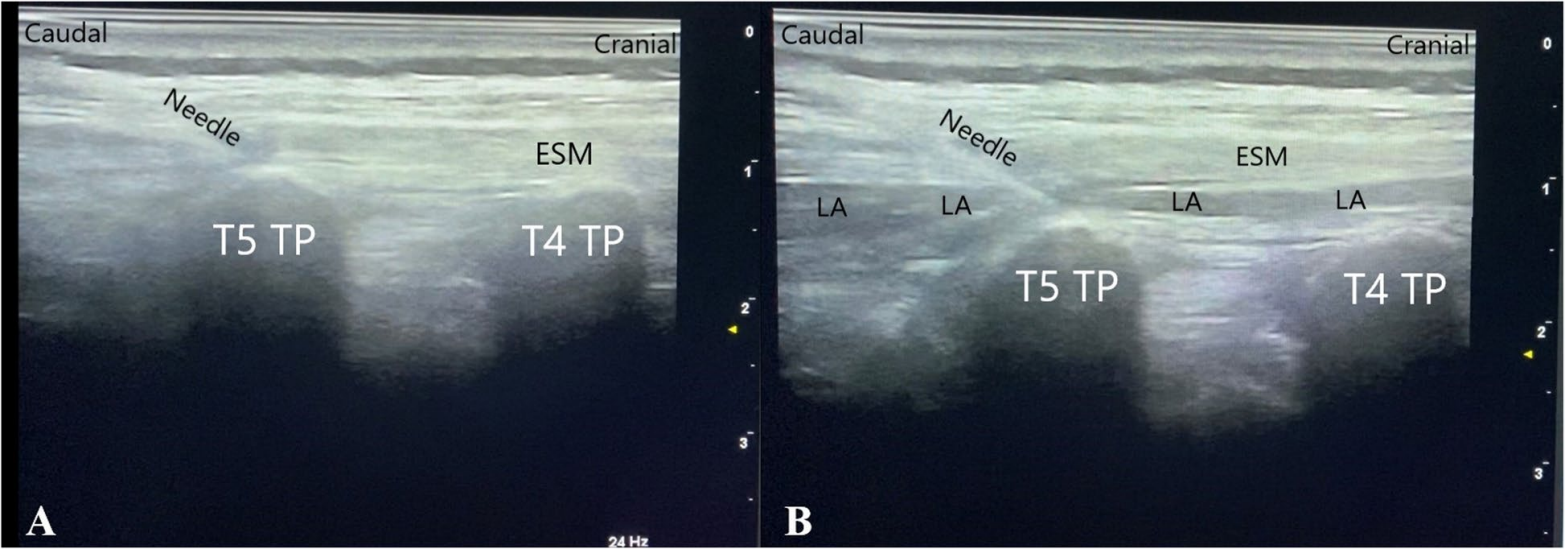
Anatomical view during Erector Spina Plane Block. **A**: The view of the block needle below the erector spinae muscle and above the transverse process. **B**: 0.25% bupivacaine was administered beneath the erector spinae muscle. The local anesthetic spread cranially and caudally beneath the erector spinae muscle

Block procedures were performed under general anesthesia before the skin incision to prevent anxiety and ensure patient comfort. Following the anesthesia induction, blocks were performed under US guidance when patients were in the lateral decubitus position. After strict skin antisepsis, the needle insertion area was covered with sterile drapes. In all patients, a high-frequency 6–18 MHz linear probe (MyLab six, Esaote, Genoa, Italy) in a sterile cover and a US-compatible 22-gauge and 8-mm nerve block needle (Pajunk, SonoPlexSTIM, Germany) were used in all groups. The following procedures were performed in the study groups. The nerve block needle was advanced with the in-plane technique under the erector spinae muscles until the interfascial space was reached. After hydrodissection with 2 ml of normal saline, 0.25% bupivacaine was injected with 30 ml in Group 1 (*n* = 30) and 20 ml in Group 2 (*n* = 30) (Fig. 1).

### Analgesia protocol

During the skin closure, patients received 50 mg of dexketoprofen and 100 mg of tramadol iv. A metoclopramide dose of 10 mg was administered iv to prevent nausea and vomiting. In the postoperative care unit, intravenous morphine was administered via a PCA pump for 24 h. Pain intensity was evaluated using a 10-point (0: No pain and 10: Unbearable pain) VAS. The PCA pump’s dose delivery was limited to administering a bolus dose of 1 mg of morphine and delivering a maximum dose of 12 mg of morphine in total within 4 h with lockout intervals of 15 min. A paracetamol dose of 1 g every 8 h and a dexketoprofen dose of 50 mg twice daily were administered iv for multimodal analgesia. As a rescue analgesic agent, 0.5 mg/kg of tramadol was given to patients iv when the VAS score at rest was ≥ 4. The patients were transferred to the ward in the 24^th^ postoperative hour. VAS scores at rest and while coughing were recorded in the postoperative 1^st^ hour, 2^nd^ hour, 4^th^ hour, 8^th^ hour, 16^th^, hour, 24^th^ hour, and 48^th^ hour. The need for additional analgesics and the presence of complications and side effects, such as hypotension, allergic reactions, respiratory depression, sedation, urinary retention, nausea-vomiting, and itching were recorded. In all patients’ data, such as age, height, body weight, BMI, gender, diagnosis, type of surgery, intraoperative and postoperative side effects, postoperative VAS scores, and postoperative additional analgesic use were recorded. The block was applied to all patients by the same anesthesiologist. VAS follow-ups of patients were performed by a pain management nurse who was blinded to the type of block applied to the patient.

### Sample size, power analysis, and statistical analysis

The sample size was calculated using G*Power© software version 3.1.9.2 (Institute of Experimental Psychology, Heinrich Heine University, Dusseldorf, Germany). The sample size was calculated for the Mann–Whitney U test, which was used for testing the main hypothesis of (VAS scores at rest in the first postoperative hour) in the preliminary study. Depending on the preliminary study research results with two-sided (two tails) type I error 0.05 and power of 80% (1-β = 0.8), effect size (d) factor 0.84, should involve ≥ 50 subjects.

The post hoc power was calculated using G*Power© software version 3.1.9.2 (Institute of Experimental Psychology, Heinrich Heine University, Dusseldorf, Germany). The power was calculated for the Mann Whitney U test, which was used for testing the main hypothesis of the present study (VAS rest first hour). Depending on previous research results with two-sided (two tails) type I error 0.05 and effect size (d) factor 1.16, post hoc power calculated as %99.99.

Data analyses were performed by using SPSS for Windows, version 22.0 (SPSS Inc., Chicago, IL, United States). Whether the distribution of continuous variables was normal or not was determined by the Kolmogorov Smirnov test. Levene test was used for the evaluation of homogeneity of variances. Unless specified otherwise, continuous data were described as mean ± SD for normal distributions, and median (Q_1_: first quartile – Q_3_: third quartile) for skewed distributions. Categorical data were described as a number of cases (%). Statistical analysis differences in normally distributed variables between two independent groups were compared by Student’s t-test, Mann Whitney U test was applied for comparisons of the not normally distributed data. Categorical variables were compared using Pearson’s chi-square test or fisher’s exact test was accepted *p*-value < 0.05 as a significant level on all statistical analysis.

## Results

**Fig. 2:**
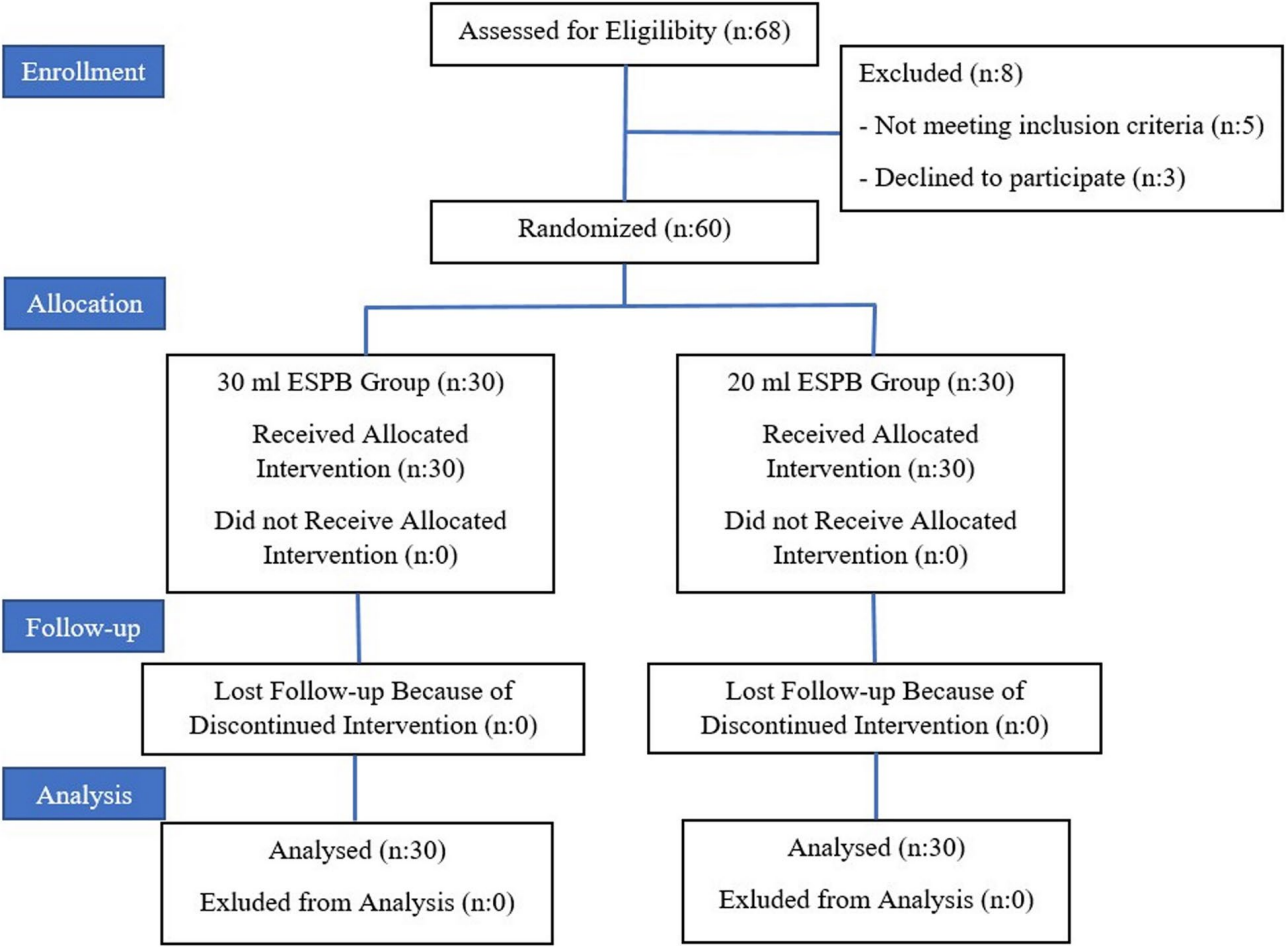
Flowchart of the patients. ESPB: Erector Spinae Plane Block

The data of a total of 60 patients who underwent thoracotomy/lobectomy between August 2021 and June 2022, were analyzed. We used CONSORT flow diagram for our study (Fig. 2).

The groups were similar in terms of demographic characteristics and surgical features (*p* > 0.05) (Table 1).

**Table 1:** Demographic characteristics and surgical features of patients

		**30 ml ESPB (** ***n*** **:30)**	**20 ml ESPB (** ***n*** **:30)**	***p***
Age, year *		51.40 ± 11,64	55.00 ± 9.45	0.194
Gender ^*δ*^	Female	12 (40.0%)	9 (30.0%)	0.417
	Male	18 (60.0%)	21 (70.0%)	
BMI ^*β*^		27.30 (23.30–28.50)	26.50 (24.20–29)	0.684
Duration of Anesthesia (minute) ^*β*^		225 (210–270)	225 (210–270)	0.868
ASA ^*δ*^	I I	12 (40.0%)	15(50.0%)	0.436
	I I I	18 (60.0%)	15(50.0%)	
Intraoperative Remifentanil Consumption (mcg)*		1049.17 ± 391.70	1013.33 ± 344.58	0.708

Continuous variables are expressed as either * the mean ± standard deviation (SD) or ^β^ the median (Q_1_: first quartile – Q_3_: third quartile), and categorical variables are expressed as either ^δ^ frequency or percentage. Continuous variables were compared with a student t-test or the Mann–Whitney U test, and categorical variables were compared using Pearson’s chi-square test or Fisher’s exact test. Statistically significant *p*-values are in bold. *BMI *Body mass index. *ASA *American Society of Anesthesiologists

No statistically significant difference was observed between the groups in terms of mean arterial pressure, heart rate, and SpO_2_ (*p* > 0.05).

**Fig. 3:**
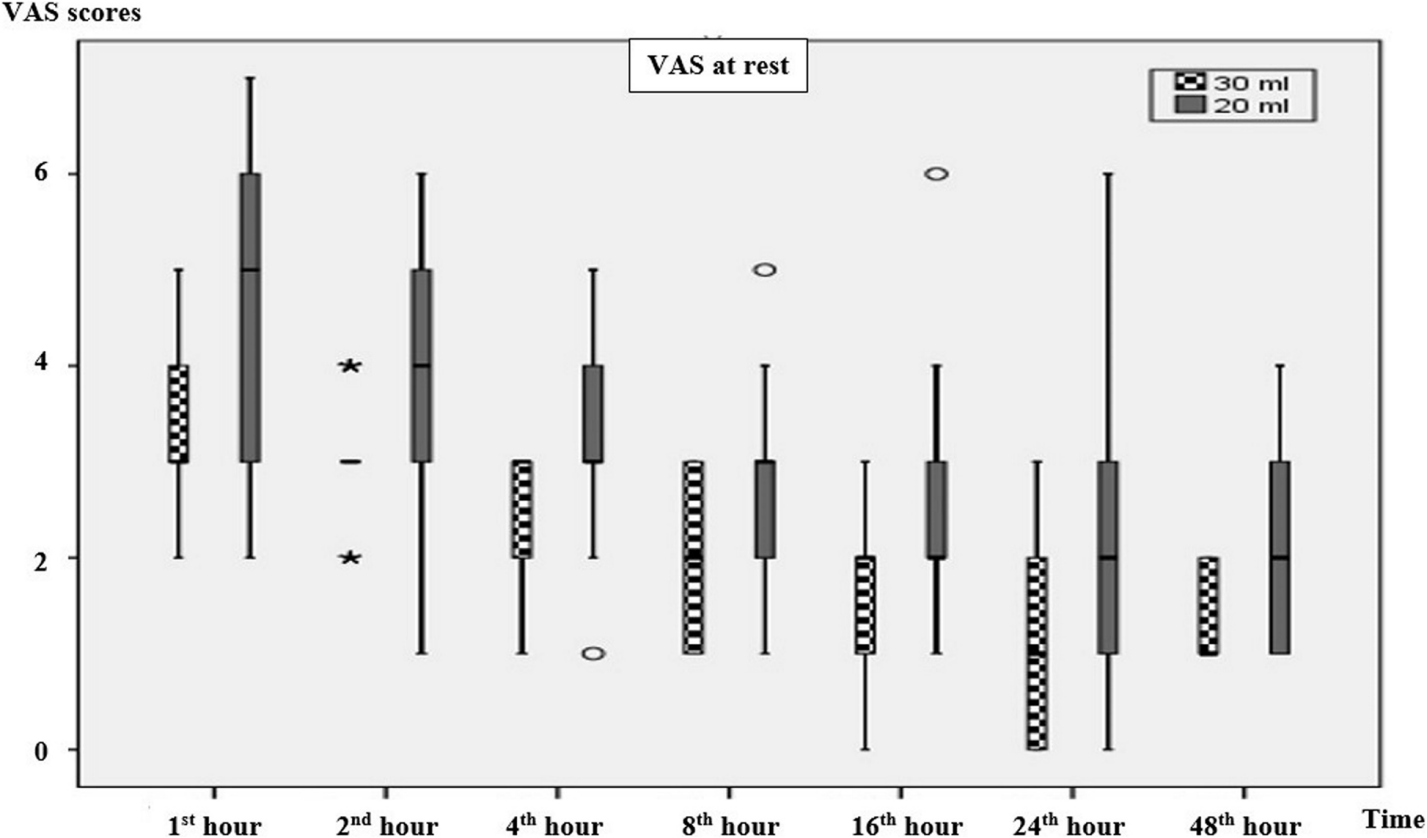
VAS scores at rest. Data are expressed as median (horizontal bars), interquartile range (boxes), and maximum and minimum values (whiskers) for the VAS scores in the 1^st^, 2^nd^, 4^th^, 8^th^, 16^th^, 24^th^, and 48^th^ hours. VAS: Visual analog scale

**Fig. 4:**
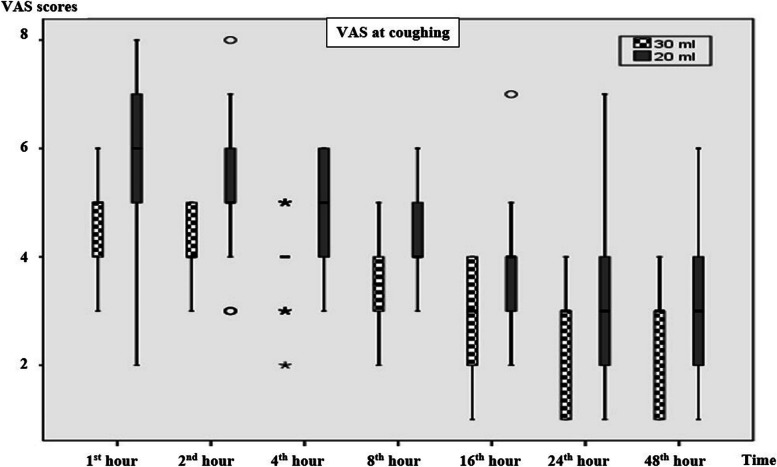
VAS scores at coughing. Data are expressed as median (horizontal bars), interquartile range (boxes), and maximum and minimum values (whiskers) for the VAS scores in the 1^st^, 2^nd^, 4^th^, 8^th^, 16^th^, 24^th^, and 48^th^ hours. VAS: Visual analog scale

When the groups were evaluated in terms of VAS resting scores, the 1^st^ (*p* = 0.001), 2^nd^ (< 0.001), 4^th^ (< 0.001), 8^th^ (< 0.001), 16^th^ (< 0.010), 24^th^ (< 0.044), and 48^th^ (< 0.005)-hour VAS resting results were found to be statistically significantly higher in the 20 ml group than the 30 ml group (Table 2) (Fig. 3). VAS coughing scores were statistically significantly higher in the 20 ml group at the 1^st^ (< 0.003), 2^nd^ (< 0.001), 4^th^ (< 0.001), 8^th^ (< 0.001), 16^th^ (< 0.004), 24^th^ (< 0.031), and 48^th^ (< 0.009) hour (Table 2) (Fig. 4).

**Table 2:** Resting and coughing VAS scores of the patients during the postoperative 48 h

	**30 ml ESPB (n:30)**	**20 ml ESPB (n:30)**	***p***
	**Med (Q**_**1**_**-Q**_**3**_**)**	**Med (Q**_**1**_**-Q**_**3**_**)**	
**VAS resting**			
20031^st^ hour	3 (3–4)	5 (3–6)	**0.001**
2^nd^ hour	3 (3–3)	4 (3–5)	** < 0.001**
4^th^ hour	3 (2–3)	3 (3–4)	** < 0.001**
8^th^ hour	2 (1–3)	3 (2–3)	** < 0.001**
16^th^ hour	2 (1–2)	2 (2–3)	**0.010**
24^th^ hour	1 (0–2)	2 (1–3)	**0.044**
48^th^ hour	1 (1–2)	2 (1–3)	**0.005**
**VAS coughing**			
1^st^ hour	5 (4–5)	6 (5–7)	**0.003**
2^nd^ hour	4 (4–5)	5 (5–6)	** < 0.001**
4^th^ hour	4 (4–4)	5 (4–6)	** < 0.001**
8^th^ hour	3.5 (3–4)	4 (4–5)	** < 0.001**
16^th^ hour	3 (2–4)	4 (3–4)	**0.004**
24^th^ hour	3 (1–3)	3 (2–4)	**0.031**
48^th^ hour	3 (1–3)	3 (2–4)	**0.009**

Continuous variables are expressed as the median (Q_1_: first quartile – Q_3_: third quartile). Continuous variables were compared with the Mann–Whitney U test. Statistically significant p-values are in bold

When the groups were evaluated in terms of morphine consumption and additional analgesic use they were found to be statistically significantly higher in the 20 ml group than in the 30 ml group (*p* < 0.001, *p* = 0.001, and *p* < 0.001, respectively) (Table 3). There was no statistically significant difference between the groups in terms of side effects (*p* > 0.05) (Table 3).

**Table 3:** Morphine consumption, additional analgesic (tramadol) use, tramadol consumption, and side effects during the postoperative 24-h need for additional analgesics, and complication rates

	**30 ml ESPB (** ***n*** **:30)**		**20 ml ESPB (** ***n*** **:30)**	***p***
Morphine Consumption (mg) ^*^	16,5 (8–22)	32 (21–40)		** < 0.001**
Additional Analgesic Use *n (%) *^*δ*^	9 (30.0%)	21 (70.0%)		**0.001**
Tramadol Consumption (mg)	50 (35–80)	120 (75–140)		**0.001**
Complication (Nausea) *n (%) *^*δ*^	3 (10.0%)	2 (6.7%)		0.999
Complication (Itching) *n (%) *^*δ*^	1(3.3%)	-		0.999

Continuous variables are expressed as either* the mean ± standard deviation (SD) or^β^ the median (Q_1_: first quartile – Q_3_: third quartile), and categorical variables are expressed as either δ frequency or percentage

## Discussion

ESPB is a newly defined regional anesthesia technique that has been used frequently in recent years. It has been reported to be beneficial in thoracic neuropathic pain as well as in acute pain after thoracic surgery or thoracic traumas [10]. To the best of our knowledge, this is the first randomized controlled trial to compare postoperative analgesia to use different local anesthetic volumes for ESPB after thoracotomy. ESPB results using 20 and 30 ml of local anesthetic in thoracotomy patients showed that the use of 30 ml of local anesthetic provided effective analgesia. In addition, similar side-effect rates show that 30 ml of local anesthetic can be used safely.

TPVB, widely used in thoracic surgery for the last 2–3 decades, has left its place in other plan blocks that can be easily applied under US guidance, as it can lead to pleural puncture and undesired neural block development [14]. For this reason, ESPB, which has become increasingly popular in recent years, is also used to prevent pain after thoracotomy. In ESPB applications, a single injection is often the preferred method. Although catheter techniques are more preferred after thoracotomy, it has been stated that a single injection technique is applied and effective analgesia is provided in the postoperative 48-h period [14, 15]. It has been stated that this analgesic effect is related to the applied multimodal analgesia [14]. In this study, ESPB using different volumes provided analgesia for 48 h after thoracotomy, while this effect had better results in the group using 30 ml local anesthetic. This situation shows that the analgesic effect can be achieved better in high volume, and also shows that multimodal analgesic technique can be an important factor.

To our knowledge, there are no studies comparing ESPB block applications using different local anesthetic volumes. However, in different studies with different volumes, it was reported that the block level increased up to 9 dermatomes in a study in which 30 ml of local anesthetic was applied [10]. In studies conducted to determine the optimal level at which volume expansion can be achieved, it has been shown that this volume varies in a wide range such as 2.5 mL/ and 6.6 mL per dermatome, while the median value is 3.4 mL [10, 16]. Çiftci et al. [17] reported that they provided effective postoperative analgesia by using 20 ml of local anesthetic in a study they conducted with US-guided ESPB for postoperative analgesia management after VATS. For acute pain control after thoracotomy, Fang et al. [14] used 20 ml of local anesthetic, while Wang et al. [18] used 30 ml of local anesthetic. Since thoracotomy is a more painful operation than VATS, which is a minimally invasive surgery, patients may need more local anesthetic volume for ESPB on acute pain after thoracotomy. Although analgesia was provided with 20 ml of local anesthetic in this study, it is important to provide more effective analgesia with 30 ml of local anesthetic in terms of creating more effective analgesia as the local anesthetic volume increases. In this respect, different volume studies will be very useful in determining the optimal volume required for ESPB.

The mechanism of action of the ESPB block, the extent of the local anesthetic and the duration of action are still controversial issues [11, 19] It is not clear how long the local anesthetic applied to the interfacial area takes effect and how long this effect lasts. Local anesthetic injected into the fascial plane must reach the nerve targets in order to create an effective block. However, how this effect occurs may differ due to the complex structure of the fascia [19–21]. The area where the ESPB block is applied is a part of the thoracolumbar fascia and this fascia consists of the thicker aponeurotic fascia. After local anesthetic injection, it is stored in this area, since the vascular structure of this part is more limited. However, local anesthetic may spread or affect these areas through the vascular and nerve structures that perforate this fascia [21, 22]. It is known that the rate of local anesthetic absorption is rapid in paravertebral and intercostal regions with high vascular structures. This variation is also seen in the fascial plane blocks, although it has not been fully clarified [23]. In a study comparing the rectus sheath block and transversus abdominal plane block, it was observed that the local anesthetic plasma peak concentration was reached in longer periods after the rectus block, and it was stated that this situation may be related to the aponeurotic fascia of the fascia structure in the area where the rectus sheath block was applied [24]. In this study, although more effective analgesia was provided in the group using 30 ml of local anesthetic, long-term analgesic effects were observed in both groups. This can be explained by the slower local anesthetic absorption, especially in fascial blocks applied to the aponeurotic area. More detailed clinical randomized studies evaluating fascial anatomy may help elucidate this mechanism.

Pain after thoracotomy is more severe and long-lasting pain due to a wider surgical incision area and trauma caused by the retractors used. For this reason, it is aimed to create a longer and continuous analgesia by applying catheter techniques. ESPB application was used to prevent pain after thoracotomy, and as in our study, single injection ESPB was frequently preferred. Fang et al. [14] in their study in which they compared the effectiveness of ESPB and TPVB, they emphasized that both blocks were effective and comparable in resting VAS scores with a single injection technique added to multimodal analgesia. In another study comparing wound infiltration and ESPB, they found that ESPB significantly reduced perioperative opioid consumption, provided better postoperative analgesia, and reduced opioid-related side effects [15]. Raft et al. [25] emphasized that ESPB performed after epidural failure in a case who underwent thoracotomy provided effective postoperative analgesia and could be a good alternative to TEA. As mentioned above, since ESPB is applied to the aponeurotic fascial area, based on the assumption that local anesthetic dissemination and elimination is slower, a single injection of local anesthetic in sufficient volume may be an alternative to epidural catheter application in thoracotomy patients.

### Limitations

This study has some limitations. First of all, a single-center planned study may not shed light on the general population for a center where very intensive thoracic surgery cases are performed. Secondly, the application was performed under general anesthesia just before the surgery in order to limit the anxiety and pain that may develop due to the block application. Therefore, the preoperative block level could not be evaluated. Third, a single injection block was applied instead of catheter application in patients. This situation could interrupt the long-term analgesia treatment that can be delivered local anesthetic by the catheter. However, considering that both multimodal analgesia application and ESPB provide long-term analgesia even if it is a single injection, we believe that an analgesic effect can be achieved. Finally, only 30 ml of local anesthetic and 20 ml of local anesthetic volumes were used for ESPB in the present study. Prospective randomized studies involving various volumes of local anesthetic may further elucidate the volume effect in ESPB.

## Conclusions

The results of ESPB applied with 20 ml and 30 ml of local anesthetic before the surgical incision in thoracotomy patients showed that the use of 30 ml of local anesthetic provided more effective analgesia. In addition, similar side-effect rates show that 30 ml of local anesthetic can be used safely. Randomized controlled studies including ESPB applications with local anesthetics to be applied in different volumes and concentrations will be beneficial in terms of providing optimal volume and revealing the mechanisms of fascial blocks.

## Data Availability

The datasets generated during and/or analyzed during the current study are available from the corresponding author on reasonable request.

## Abbreviations

ASA: American Society of Anesthesiologists
BMI: Body mass index
ESPB: Erector spinae plane block
PCA: Patient-controlled analgesia
TEA: Thoracic epidural analgesia
TPVB: Thoracic paravertebral block
US: Ultrasound
VAS: Visual analog scale
