# Correction: Comparison of analgesic efficacy of different local anesthetic volumes for erector spinae plane block in thoracotomy patients; a prospective randomized trial

**DOI:** 10.1186/s12871-023-02031-1

**Published:** 2023-03-07

**Authors:** Musa Zengin, Hilal Sazak, Ramazan Baldemir, Gulay Ulger, Dilara Arican, Oya Kaybal, Ali Alagoz

**Affiliations:** Anesthesiology and Reanimation Clinic, University of Health Sciences, Ankara Atatürk Sanatorium Training and Research Hospital, Kuşcağız Mah. Sanatoryum Cad. No: 271, Ankara, Turkey

Correction: BMC anesthesiol 23, 42 (2023)10.1186/s12871-023-02004-4

Following the publication of the article [1], it was noticed that there was a lack of expression and a citation error.

In the first paragraph of the discussion section of the article, there is the statement ‘To the best of our knowledge, this is the first randomized controlled trial to compare postoperative analgesia to use different local anesthetic volumes for ESPB after thoracotomy’. However, in the third paragraph of the discussion section, there is the statement ‘To our knowledge, there are no studies comparing ESPB block applications using different local anesthetic volumes’. This second statement did not specify that ESPB application covers thoracotomy patients. The correct expression should have been ‘To our knowledge, there are no studies comparing ESPB block applications using different local anesthetic volumes after thoracotomy’. However, Abdella et al [2] compared different volumes for ESPB after mastectomy and Altıparmak et al [3] analyzed 2 different concentrations of bupivacaine in mastectomies.

A citation error was made in the same paragraph of the discussion section. The studies of Forero et al. were mixed. Then, the citation was made to another article by Forero et al.

Correctly cite ‘To our knowledge, there are no studies comparing ESPB block applications using different local anesthetic volumes after thoracotomy. However, in a case series with different volumes, it was reported that the block level increased up to 9 dermatomes in a case in which 30 ml of local anesthetic was applied [4]. In studies conducted to determine the optimal level at which volume expansion can be achieved, it has been shown that this volume varies in a wide range such as 2.5 mL/ and 6.6 mL per dermatome, while the median value is 3.4 mL [5]’ should be in the form.

We apologize for any inconvenience caused by this error.
